# Virtual splint registration for electromagnetic and optical navigation in orbital and craniofacial surgery

**DOI:** 10.1038/s41598-021-89897-8

**Published:** 2021-05-17

**Authors:** Ruud Schreurs, F. Baan, C. Klop, L. Dubois, L. F. M. Beenen, P. E. M. H. Habets, A. G. Becking, T. J. J. Maal

**Affiliations:** 1 Department of Oral and Maxillofacial Surgery, Amsterdam UMC Location AMC and Academic Centre for Dentistry Amsterdam (ACTA), University of Amsterdam, Meibergdreef 9, 1105 AZ Amsterdam, The Netherlands; 2 Department of Oral and Maxillofacial Surgery 3D Lab, Radboud University Medical Centre Nijmegen, Radboud Institute for Health Sciences, Geert Grooteplein-Zuid 10, 6525 GA Nijmegen, The Netherlands; 3 Department of Radiology and Nuclear Medicine, Amsterdam UMC Location AMC, University of Amsterdam, Meibergdreef 9, 1105 AZ Amsterdam, The Netherlands; 4 Department of Medical Biology, Section Clinical Anatomy and Embryology, Amsterdam UMC Location AMC, University of Amsterdam, Meibergdreef 9, 1105 AZ Amsterdam, The Netherlands

**Keywords:** Preclinical research, Three-dimensional imaging

## Abstract

In intra-operative navigation, a registration procedure is performed to register the patient’s position to the pre-operative imaging data. The registration process is the main factor that determines accuracy of the navigation feedback. In this study, a novel registration protocol for craniofacial surgery is presented, that utilizes a virtual splint with marker points. The accuracy of the proposed method was evaluated by two observers in five human cadaver heads, for optical and electromagnetic navigation, and compared to maxillary bone-anchored fiducial registration (optical and electromagnetic) and surface-based registration (electromagnetic). The results showed minimal differences in accuracy compared to bone-anchored fiducials at the level of the infra-orbital rim. Both point-based techniques had lower error estimates at the infraorbital rim than surface-based registration, but surface-based registration had the lowest loss of accuracy over target distance. An advantage over existing point-based registration methods (bone-anchored fiducials, existing splint techniques) is that radiological imaging does not need to be repeated, since the need for physical fiducials to be present in the image volume is eradicated. Other advantages include reduction of invasiveness compared to bone-achnored fiducials and a possible reduction of human error in the registration process.

## Introduction

In image-guided surgical navigation, registration is a prerequisite to determine the position of a surgical navigation instrument relative to the patient’s position and visualize this position in the pre-operative imaging data^1–5^. The coordinate system associated with the pre-operative imaging data is registered to the position of the dynamic reference frame and thus the position of the patient’s anatomy within the OR. The quality of the registration process has been widely regarded as the key factor that determines navigation accuracy and safety^1,6–13^.

The amount of registration techniques available has expanded throughout the years, and different categorizations between registration methods can be made based on invasiveness (invasive vs. non-invasive^1,14,15^). Invasive registration procedures are more cumbersome because of fiducial placement and pose a (minimal) risk to the patient, but are associated with a higher accuracy^1,6^. Other possible categorizations are the dependency on marker points (marker-based vs. marker-less^7,9,10,16^), underlying registration algorithm (pair-point vs. surface-based^1,5,6,10,16,17^) or user-dependency of the process^1,18,19^. The choice for a registration technique is dependent on accuracy requirements (lateral skull base surgery in Otolaryngology requires submillimeter precision only at the lateral skull base, neurosurgical navigation requires accurate localization in deep structures), invasiveness of fiducial placement in relation to the invasiveness of surgery (bone-anchored fiducials are contra-indicated in non-invasive surgical procedures), soft-tissue deformation (swelling due to facial trauma may hamper accurate surface-based registration) and pre- or intra-operative logistics (time between imaging and surgery, availability of intra-operative auto-registration modules).

In oral and maxillofacial surgery, intra-operative navigation is mainly used in midfacial traumatology and orbital reconstruction^3,20,21^. Bone-anchored fiducials may be positioned intra-orally if possible, to ease the insertion and reduce scar visibility. Splint-based registration, where fiducial marker points are attached to a maxillary dental splint, provides a non-invasive alternative to bone-anchored fiducials, with sufficient accuracy for navigation in the midface^7,9,22^. These pair-point registration methods require the presence of fiducials in the image data, usually a computed tomography (CT) or cone-beam computed tomography (CBCT) scan. Frequently, this means acquisition of a second registration scan in addition to the diagnostic scan is required, with the fiducial markers in place. This may pose logistical challenges, especially in bedridden patients, and gives additional exposure to radiation for the patient.

In this study, a novel non-invasive splint-based registration method without the necessity of additional radiologic imaging is proposed: virtual splint registration. The workflow for virtual splint registration is detailed in the “Methods” section. A cadaver study was performed with the aim to evaluate the accuracy of the novel registration method for optical and electromagnetic (EM) navigation, and compare it to the accuracy of maxillary bone-anchored fiducial registration (optical and EM) and soft-tissue registration (EM).

## Methods

### Cadaver set-up

**Figure 1:**
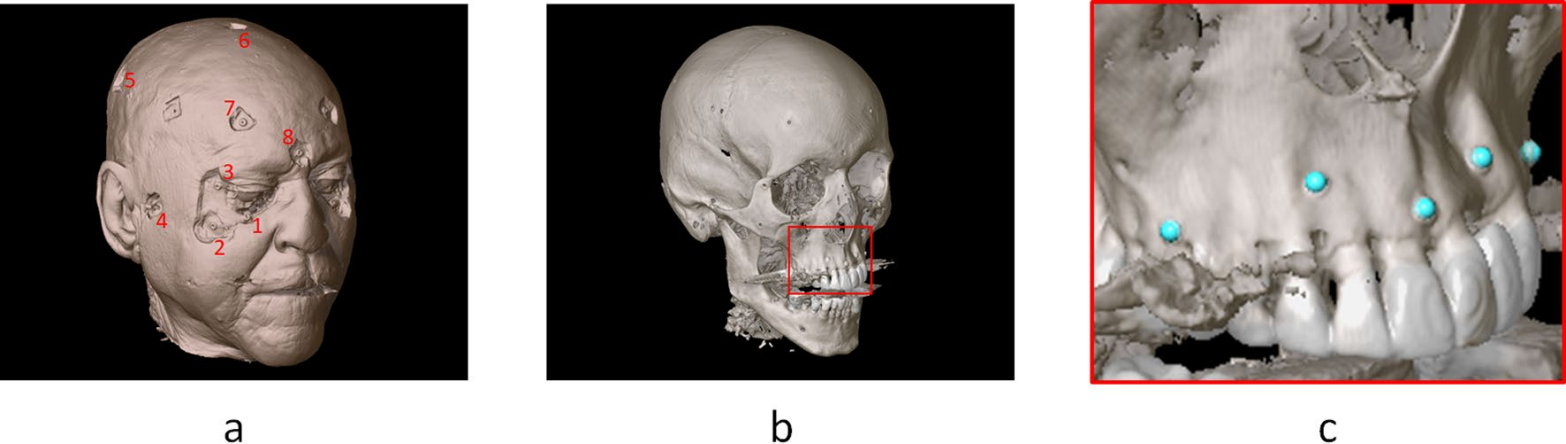
Cadaver model. The numbers at the Allen screw positions (**a**) correspond to the anatomic landmarks provided in Table 1. In (**b**), the titanium screws in the maxilla are visualized; in (**c**), the registration points have been indicated in the screw head.

Five human cadaver heads were obtained through the body donation program from the Department of Medical Biology, Section Clinical Anatomy and Embryology of the Amsterdam UMC (location AMC). The bodies from which the samples were taken were donated to science in accordance with Dutch legislation and the regulations of the medical ethical committee of the Amsterdam UMC. The experimental protocol was approved by the review committee of Medical Biology, Section Clinical Anatomy and Embryology (ref. 2018-087). All methods were performed in accordance with the relevant guidelines and regulations. The heads were fixated in a phenol (0.4% w/v), methanol (1.3% w/v), formaldehyde (4.8% w/v), glycerine (10.3% w/v), ethanol (24.8% w/v) solution in water and preserved in a phenol (0.21% w/v), ethanol (8.3% w/v), glycerol (16.7% w/v) solution in water. The cadaver heads had been previously used in another study; the edentulous cadavers and the cadaver with restorations on all maxillary elements were excluded^23^. The dental status of the maxillary dentition is summarized in Table 1. Fourteen Poly-Ether-Ether-Ketone Allen screws (M3, 10 mm, Jeveka B.V., Almere, The Netherlands) were fixated to serve as target fiducials to evaluate the accuracy of the registration (Fig. 1a). The position of these target fiducials corresponded to the landmarks provided in Table 2 as closely as possible. This particular type of screw was chosen because of the indentation in the floor of the hexagonal opening, providing a highly reproducible target for the navigated surgical instrument, as well as the absence of image artefacts associated with metallic screws, easing target identification in the image data. Five titanium screws (1.5 × 5.0 mm maxDrive screws, KLS Martin, Tuttlingen, Germany) were fixated on the maxilla to serve as bone-anchored fiducial points, mimicking current clinical protocol (Fig. 1b,c). An optimal distribution of the screws was sought, avoiding fixation near dental roots.

**Table 1:** Dental status of the cadavers.

Cadaver	Decayed	Missing	Span	Filled	Metal filling	Composite filling
1	–	–	17–27	12	4	8
2	8	5	17–23	–	–	–
3	–	9	14–23	1	1	–
4	–	–	17–27	1	-	1
5	2	4	17–27	2	2	-

The DMFT status is complemented with span of the dental arch (most distal element in both quadrants) and type of filling.

**Table 2:** Anatomical landmarks positions.

Number	Landmark name	Bilateral
1	Infraorbital rim	Yes
2	Zygomatic prominence	Yes
3	Frontozygomatic suture	Yes
4	Porion	Yes
5	Temporal bone	Yes
6	Cranium	No
7	Frontal bone	Yes
8	Nasion	No

The numbers correspond to the numbers in Fig. 1a.

### Imaging

**Figure 2:**
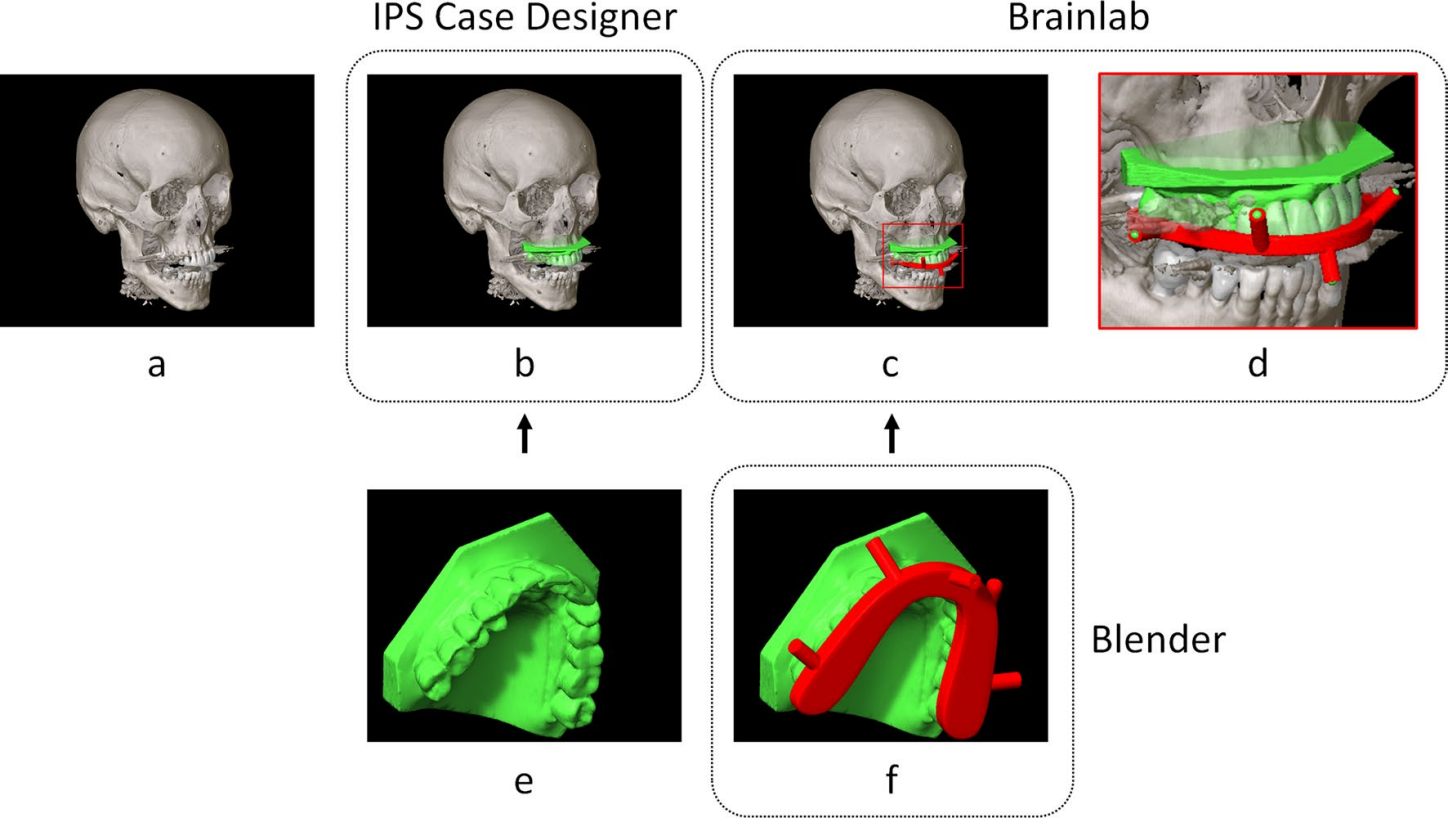
Virtual splint registration workflow. The computed tomography scan (**a**) and the intra-oral scan (**e**) are fused in IPS case designer software (**b**). In blender, a navigation splint is designed with five cylinders with an indentation augmented on it (**f**). The dental model and splint are imported in the Brainlab environment (**c**): the dental model for control purposes and the navigation splint to indicate the registration points (**d**).

A pre-operative CT scan (Siemens Somatom Force, Siemens Healthineers, Forchheim, Germany) was acquired with the following scan parameters: 120 kV, 37 mA, collimation 0.6 mm, pitch 0.85, FOV diameter 25 cm, matrix size 512 × 512, slice thickness 0.6 mm, bone kernel Ur77 (Fig. 2a). An intra-oral scan of the maxillary dentition (TRIOS 3 intraoral scanner, 3Shape, Copenhagen, Denmark) was acquired according to the scanning protocol provided by the manufacturer (Fig. 2e). The DICOM data were imported in IPS CaseDesigner (version 1.4, KLS Martin, Tuttlingen, Germany), a software environment specific for virtual surgical planning of orthognathic surgery cases. The intra-oral scan was automatically fused to the hard-tissue patient model, to replace the low definition dental information of the (CB)CT with a highly detailed dental surface model (Fig. 2b)^24^. The algorithm uses contrast information at the tooth crown margin to perform the fusion. The accuracy was visually verified in the software; if necessary, a sequential fusion could be performed.

### Splint design

The fused dental model was exported as stereolithographic models (stl) and imported in Blender (version 2.81, Blender Foundation, Amsterdam, the Netherlands). An offset of 0.1 mm was given to the dental model for splint creation, to ensure proper fit of the splint. In Blender, a virtual maxillary splint was designed; the splint was augmented with five cylinders, 10 mm in length and 4.5 mm in width. The cylinders were as widely distributed on the splint as feasible (Fig. 2f). At the protruding end of each cylinder, an indentation had been created in the shape of a hemisphere (diameter 1 mm) to serve as a virtual fiducial landmark. The splint was exported in stl format, and 3D printed with an in-house Polyjet printer (Objet30 Prime, Stratasys Ltd., Eden Prairie, MN, USA) in transparent material (VeroClear).

### Navigation planning

The CT scan was imported in the Brainlab planning environment (Origin/iPlan version 3.0.6, Brainlab AG, Munich, Germany). The heads of the bone-anchored maxillary screws were indicated as registration fiducials (Fig. 1c). A landmark was positioned on every target screw. Although the virtual patient model was built from the same DICOM information, the Brainlab software environment uses a different image reference frame than the IPS software environment. The virtual model of the registration splint needed to be transformed to the corresponding Brainlab position using the algorithm provided below:

$$\left[\begin{array}{c}{X}_{Brainlab}\\ {Y}_{Brainlab}\\ \begin{array}{c}{Z}_{Brainlab}\\ 1\end{array}\end{array}\right]=\left[\begin{array}{cccc}-1& 0& 0& -{X}_{IPP}\\ 0& -1& 0& -{Y}_{IPP}\\ 0& 0& 1& {Z}_{IPP}\\ 0& 0& 0& 1\end{array}\right]*\left[\begin{array}{c}{X}_{IPS}\\ {Y}_{IPS}\\ \begin{array}{c}{Z}_{IPS}\\ 1\end{array}\end{array}\right]$$

Th subscript IPS indicates the IPS coordinate system, Brainlab indicates the Brainlab coordinate system and IPP indicates ImagePositionPatient in the dicom header of the first dicom slice. The transformed reference splint model was imported in the Brainlab environment using the STL Import function (Fig. 2c). Five registration points were easily indicated on the base of the indentations of the cylinders (Fig. 2d). The planning was exported in DICOM format from the Origin environment. The coordinate of the exact vertex corresponding to the pole position of the sphere used to create the indentation was known for each cylinder. The position of the indicated registration points was fine-tuned through manipulation of the DICOM data: the manually indicated coordinates were replaced by the exact coordinate of the indentation.

### Accuracy measurements

The DICOM planning data corresponding to the specific cadaver head was imported in the Kick navigation system (Brainlab AG, Munich, Germany), a system compatible with both optical and electromagnetic tracking. For EM tracking, the reference array was ridigly fixated to the cadaver’s skull. The field generator was positioned laterally. In the ENT module of the Kick system, both soft-tissue registration (surface-based) and point-based registration could be performed with the EM registration pointer. The surface registration was performed according to the instructions provided by the manufacturer: after initialization with three anatomical landmarks (exocanthion left and right, nasion), the pointer was moved along the cheeks, forehead and the nasal ridge in a sinusoidal fashion. Point-based registration on the bone-anchored maxillary screws was performed by positioning the EM registration instrument in the cross of the screws’ heads and clicking the button on the instrument. For splint-based registration, the splint was positioned by the observer and held in place on the dentition; the indentations of the five cylinders were indicated with the registration instrument.

In the optical navigation setting, the craniomaxillofacial (CMF) module was used. Only point-based registration was available. The Reference Base Skull (Brainlab AG, Munich, Germany) was fixated to the cadaver’s skull and the Skull Reference Array (Brainlab AG, Munich, Germany) was attached. The optical tracker camera was positioned close to the surgical site, while still in the optimal range, indicated in the Camera view. The Cranial/ENT Pointer (two reflective markers) was used in the optical setting. Point-based registration was performed by positioning the tip of the pointer on the fiducial landmark and pivoting the instrument. Two observers (RS, FB) each performed five repetitions for each combination of registration method and tracking technique: in total, 50 registrations were performed on each cadaver [soft-tissue registration (EM): 10, bone-anchored maxillary screws (EM): 10, bone-anchored maxillary screws (optical): 10, splint-based (EM): 10, splint-based (optical): 10]. After each registration, all fourteen Allen screws were indicated with the instrument and their coordinate position as provided by the navigation system was stored using the Acquire-functionality. The landmarks indicated on the Allen screws in the image volume were not imported in the Kick system, since the distance quantification provided by the navigation system, between a landmark and the pointer’s position, could affect the observer’s independence to the outcome of the measurement.

### Data analysis

After each observer performed the repeated registrations and measurements, the data was saved and exported in DICOM format. The coordinates of the acquired landmarks were extracted from the DICOM data in Matlab (version 2019b, the MathWorks Inc., Natick, MA, USA). The target coordinates were extracted in a similar fashion from the planned DICOM set. In Matlab, the Euclidean distance between the acquired coordinate and the target coordinate was calculated for each landmark measurement (Target Registration Error, TRE). The distance between each target landmark and the center of gravity of the fiducial landmarks was calculated to serve as a measure for target distance to the registration area. The data was imported in Rstudio (version 1.2.5033, RStudio, Inc., Boston, MA, USA) for statistical analysis^25^. For assessment of interobserver correlation, the mean of each repeated measurement (same observer, landmark, registration method, and tracking technique) was calculated; the intraclass correlation coefficient (absolute agreement) was calculated between these measurements for observer 1 and observer 2. An ICC value < 0.5 was considered poor agreement between observers, a value between 0.5 and 0.75 was considered moderate agreement, between 0.75 and 0.90 good agreement and ≥ 0.90 excellent agreement. The distribution of the data was evaluated with histograms and kernel density estimate plots. A transformation of the data would be applied if necessary. A linear mixed model approach was used, since several factors may have had an effect on the accuracy^26^. Distance to the registration center, tracking technique and registration method, as well as their interactions, were included as fixed effects; cadaver was programmed as a random effect. The p value is not provided by the ‘lme4’-package since an exact calculation of the p value is not feasible in the mixed model approach. An approximation of the p value was obtained for the fixed effects with the ‘lmerTest’-package^27^.

## Results

**Figure 3:**
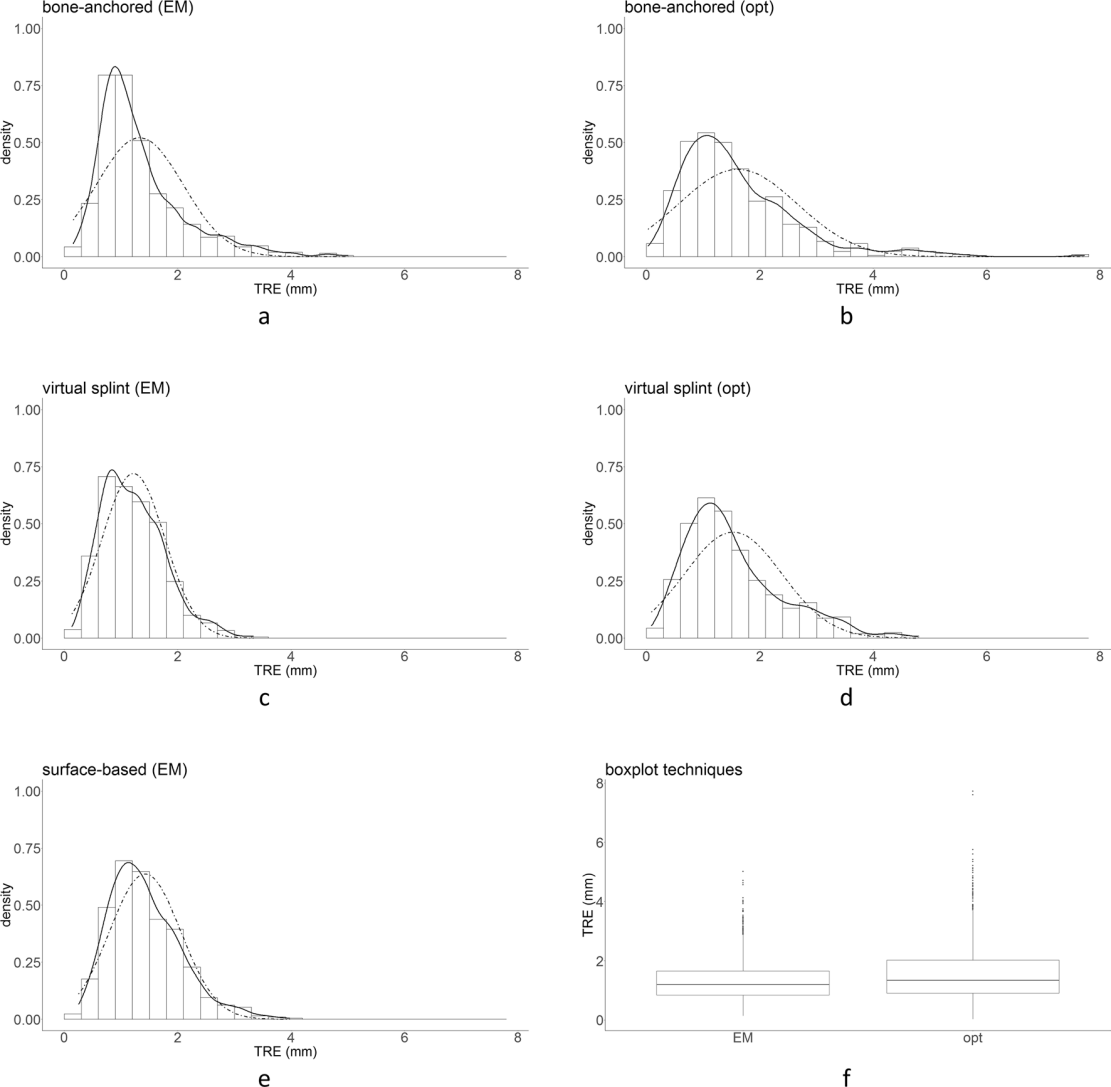
Distribution of TRE for each combination of tracking technique and registration method in histogram and density estimation plots (**a**–**e**). A normal distribution corresponding to the mean and standard deviation of the data is visualized as a dotted curve. The data is skewed to the right. In (**f**), box and whisker plots per registration technique are visualized, which point to right-skewed data as well.

**Figure 4:**
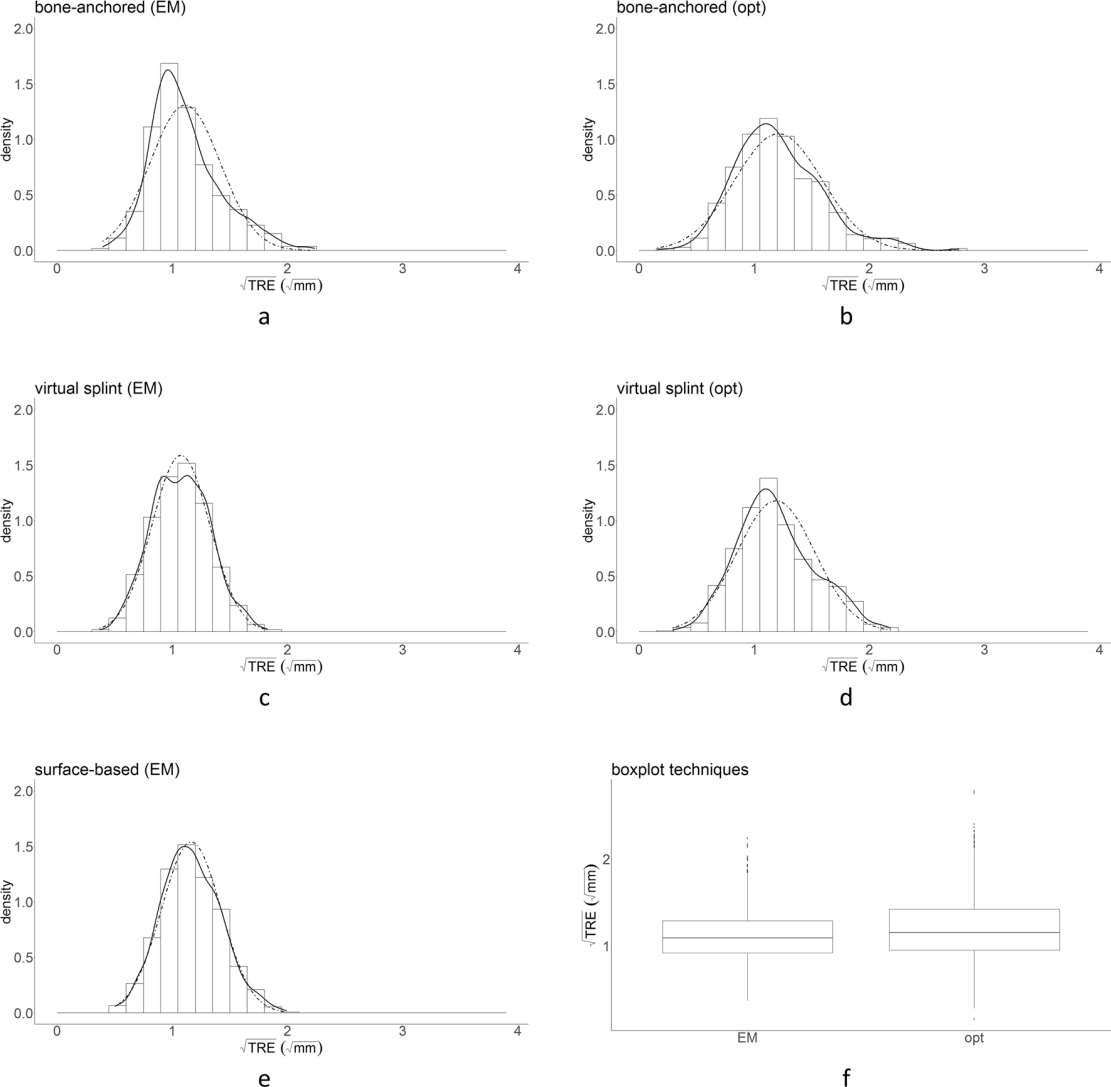
Distribution of $\sqrt{\mathrm{TRE}}$ for each combination of tracking technique and registration method (**a**–**e**), and the box and whisker plots (**f**). The distribution of the data corresponds better to a normal distribution after the data correction.

In total, 3483 data points were collected [17 (0.5%) were missing]. In Fig. 3, histograms are presented for each combination of tracking technique and registration method; density estimations and a normal distribution corresponding to the mean and standard deviation of the TRE are added. A box and whisker plot for the TRE per tracking technique is shown in Fig. 3f. An ICC of 0.52 between the observers was found, indicating moderate agreement; the mean difference between the observers was 0.11 mm. Based on the findings in the histograms, kernel density estimate plots and box and whisker plot, a data transformation was applied to the TRE outcome variable to correct the skewedness of the data and meet the normality assumption for linear mixed model analysis. A square root transformation of TRE was performed; the distribution of $\sqrt{TRE}$ is visualized in Fig. 4. A correction of the skewedness and better fit to a normal distribution can be observed from the histograms, density estimated plots and box and whisker plots in Fig. 4.

**Figure 5:**
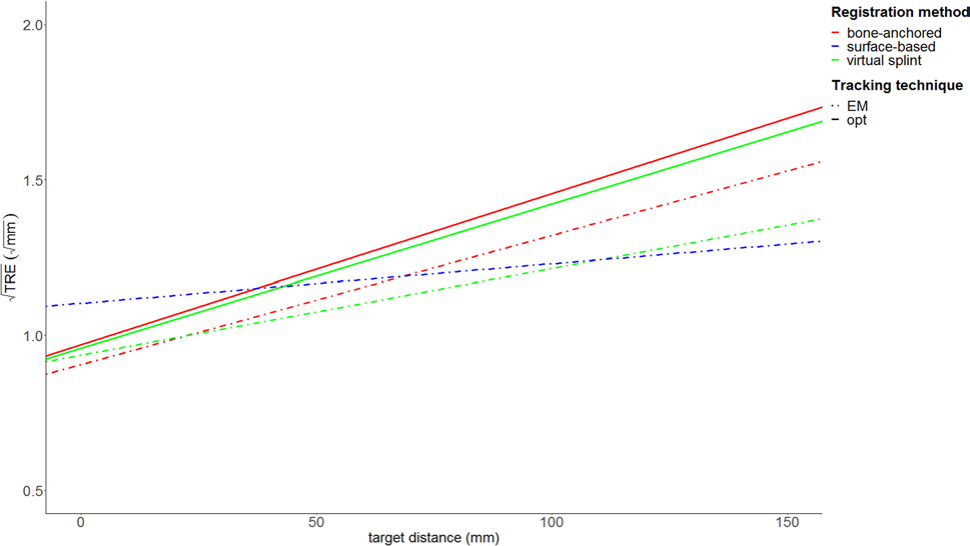
Plot of target distance against $\sqrt{\mathrm{TRE}}$ for each combination of tracking technique and registration method. The plot corresponds to the slope and intercept values presented in Table 2.

The distance variable was transformed by subtracting the mean distance of the orbital landmark in all cadavers. This was done to restrict the prediction range of the model to actual landmark positions and to give meaning to the fixed effect intercept estimation of the model: the intercept value at distance zero corresponds to the $\sqrt{TRE}$ estimation at the mean infraorbital rim level of the five cadavers. The fixed-effect estimates, standard deviations and t values are listed in Table 3. The Wald statistic (t value) provides a scaled effect size for each parameter. In the model output, the fixed effect estimates are provided as increment or decrement relative to the reference intercept, which was bone-anchored registration with optical navigation. In Table 4, the relative values are recalculated to provide two absolute values for each combination of tracking technique and registration method: an intercept value, which indicates the value of $\sqrt{TRE}$ at the level of the infraorbital rim (as stated above) and a slope value, to indicate the effect of distance on the TRE value (increase of $\sqrt{TRE}$ per mm). A plot based on the intercept values of $\sqrt{TRE}$ and the slope for distance increase is visualized in Fig. 5, with distance (transformed as stated above) on the x-axis and $\sqrt{TRE}$ on the y-axis.

**Table 3:** Fixed-effect estimates.

Fixed effect	Estimate	Standard dev	t value	p value
(Intercept)	0.970	0.0348	27.86	< 0.001
Distance	+ 0.005	0.0002	23.07	< 0.001
EM technique	− 0.064	0.0202	− 3.19	0.001
Virtual splint registration	− 0.011	0.0203	− 0.55	0.58
Surface-based registration	+ 0.197	0.0202	9.78	< 0.001
Distance:EM technique	− 0.001	0.0003	− 2.35	0.02
Distance:virtual splint registration	+ 0.000	0.0003	− 0.72	0.47
Distance:surface-based registration	− 0.003	0.0003	− 9.73	< 0.001
EM technique:virtual splint registration	+ 0.042	0.0286	1.47	0.14
Distance:EM technique:virtual splint registration	− 0.001	0.0004	− 2.74	0.006

Bone-anchored registration with optical navigation was used as the reference category.

**Table 4:** Intercept and slope value estimates for each combination of tracking technique and registration method.

Tracking technique	Registration method	Intercept	Slope
Optical	Bone-anchored fiducials	0.97	0.0049
Optical	Virtual splint	0.96	0.0046
Electromagnetic	Bone anchored fiducials	0.91	0.0042
Electromagnetic	Virtual splint	0.94	0.0028
Electromagnetic	Surface-based	1.10	0.0013

**Figure 6:**
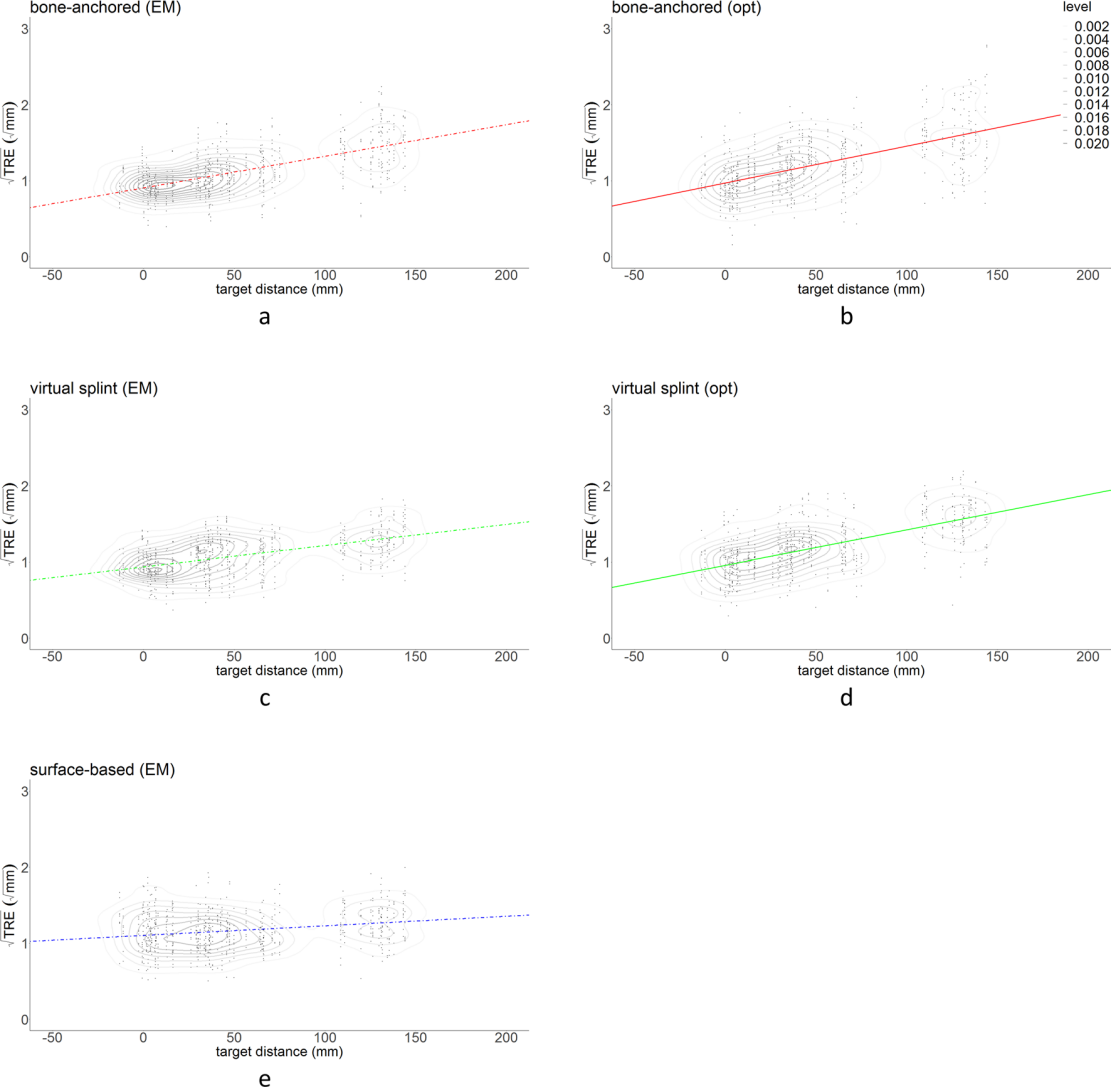
Combined scatter plots and kernel density estimates for each registration method and tracking technique, for all data points. From the kernel density estimate levels, the concentration of measurements is visualized. The corresponding regression line from Fig. 3 is superimposed on the scatter plot.

A combined scatter and kernel density estimate plot is presented for each technique/registration combination in Fig. 6, and the regression line from Fig. 5 is added to the data distribution. From the scatter plots, it can be seen that the distribution of $\sqrt{TRE}$ is more spread out for bone-anchored fiducials than it is for the virtual fiducial registration technique, both for optical and electromagnetic tracking; the surface-based registration measurements seem more concentrated as well.

## Discussion

In this study, a novel non-invasive registration method for intra-operative navigation, using fiducial landmarks on a virtual splint, was presented. The accuracy was tested in a cadaver set-up using optical and electromagnetic tracking, and compared to maxillary bone-anchored screw registration and soft-tissue surface-based registration. The ICC of 0.52 showed moderate agreement between the observers. This indicates that differences between the observers are relatively large compared to the variance within the population. The repeated-measures design with two observers and multiple repetitions per observer was chosen to be able to assess the accuracy of each registration approach as meticulously as possible. The moderate observer agreement may warrant some caution in extrapolation of the results, but the difference of 0.11 mm is not expected to have a significant biasing effect. On the level of the infraorbital rim, virtual splint-based registration was slightly more accurate than the bone-anchored fiducials for optical navigation, but slightly less accurate for the EM navigation. Both point-based methods were significantly more accurate than surface-based registration at the level of the infraorbital rim. The accuracy of the registration was reduced for landmarks farther from the registration area; the loss of accuracy over distance was reduced for splint-based virtual fiducial registration compared to bone-anchored fiducial registration. This may be because of the larger dispersal between the splint fiducials compared to the bone fiducials. The distance-$\sqrt{TRE}$ slope was lowest for surface-based registration, which means that surface-based registration is less prone to loss of accuracy over distance. The $\sqrt{TRE}$ estimate of the point-based methods was < 1.0 at the level of the orbital rim, but > 1.4 at a distance of 100 mm from the orbital rim (away from the registration center) for optical navigation. The percentage of TRE measurements > 2 mm ($\sqrt{TRE}$ > $\sqrt{2}$) was 27% for optical bone-anchored fiducials, 24% for optical virtual fiducials, 15% for electromagnetic bone-anchored fiducials, 9% for electromagnetic virtual fiducials and 17% for electromagnetic soft-tissue registration. Overall, EM tracking seemed more accurate than optical tracking in this study.

Several errors can affect the accuracy of the navigation process intra-operatively: a subdivision into technical errors, imaging errors, registration errors, application errors and human errors was proposed by Widmann et al.^1^. Three registration errors can be distinguished in point-based registration: the fiducial localization error (FLE), the fiducial registration error (FRE) and the target registration error (TRE)^6,17,28,29^. The FLE is defined as the error in locating the exact position of a fiducial point, which is made up of the identification error in the image data (FLE_im_) and intra-operatively in the physical space (FLE_ph_)^8^. In the registration process, the pre-operatively annotated image fiducials are as closely aligned as possible with the patient fiducial points indicated by the surgeon; the FRE is defined as the root mean square distance between the corresponding fiducials that exists after registration. The TRE is the most interesting measurement: this is defined as the discrepancy between the actual location of the instrument and the visualized location of the instrument for a landmark not used as a registration fiducial. The TRE is proportional to the FLE, but also dependent on configuration of the fiducials and distance to the fiducials’ center^1,6,29^.

Splint-based registration methods have been extensively proposed and studied in literature as a non-invasive alternative to bone-anchored screws^7,9,10,15,22,30–35^. Radiopaque markers that can be recognized in the (CB)CT scan, like titanium screws, gold beads or gutta-percha markers, can be fixated to the splint. Designs with extenders have been proposed for alternative configurations of the fiducials, either to enhance the distribution or to center them around the target area, reducing the distance between target and fiducials’ center^9,15,32–34^. The common denominator among all splint-based techniques is the necessity to acquire imaging with the splint in position. Next to the previously mentioned adverse effect of repetitive imaging, other drawbacks are associated with this method. The registration is dependent on the similarity of splint positioning in the OR to the scan position; a discrepancy in splint positioning will reduce registration accuracy^7,9^. The fiducials need to be indicated on the scan volume manually and the FLE_im_ may increase due to image artifacts originating from the fiducials themselves, or dental restorations in the vicinity. Finally, positioning of the splint before the scan may be forgotten, rendering the scan useless for navigation^7,32^.

The virtual splint method eliminates some of these error sources: the manual identification of fiducials can be automated and the splint is positioned only once, in the OR. In the design of the splint, the distribution configuration of the fiducials can be optimized and in theory, extenders are even possible. Other sources of fiducial localization error may arise in the workflow. The FLE_ph_ is dependent on an exact fit of the splint according to the virtual planning, which in turn is dependent on the design of the splint and accuracy of the printing process. In an extended design, stiffness of the material will also have an increased effect on fiducial localization during surgery^9^. The FLE_im_ is now dependent on the fusion accuracy of the intra-oral scan on the position of the dentition in the (CB)CT. The fusion to create a virtual patient model with high definition dentition, and the virtual design and production of splints is common practice in orthognathic surgery and considered reliable for feedback during surgery. Further optimization of the design of the splint, improvements in metal artifact reduction in CT imaging and sophisticated algorithms for registration of intra-oral scans to CT may have a positive effect on the accuracy of the proposed workflow.

Quantification of the TRE in the clinical setting is infeasible: fixation of additional bone-anchored fiducials just for measurement purposes is unethical, while measuring at anatomical landmark positions will be inaccurate due to the large difference in localizing the exact anatomical position in the image volume and in the OR (target localization error, TLE^28,29^). Several ex-vivo methods have been proposed for registration accuracy studies, such as phantom heads^4,7,9,11,12,16^, 3D-printed skull models^13–15,36^, cadaver bone preparations^37,38^ or cadaver heads^2,39^. The rationale for a cadaver set-up in this study was to mimick the clinical setting as accurately as possible and to include all sources of error associated with the novel protocol: FLE_im_ could only be included by performing the registration of the intra-oral scan on an actual dentition. Care should be taken with the interpretation of the surface-based matching results in a set-up with fixated cadavers: soft tissue changes due to swelling or difference in facial expression encountered in clinical practice are not accounted for. Another limitation of the cadaver model was the presence of multiple restorations in some of the cadaver heads, which may have affected accuracy of the intra-oral scan registration. The differences in ex-vivo method, position of bone-anchored fiducials and imaging protocol makes one-to-one comparison to results in literature infeasible. The accuracy study on splint registration described by Luebbers et al. and Venosta et al. is most closely related to the current study, although in these studies a synthetic skull model was used^9^. Nevertheless, the results from the virtual fiducial splint registration are in accordance with the findings by Luebbers et al. and Venosta et al.^7,9^.

Based on the results of the accuracy study, virtual splint-based registration is a valuable alternative to bone-anchored maxillary screws for surgical navigation in the midface and an excellent alternative to splint-based registration methods that require the splint to be in position during scan acquisition. Advantages of the proposed registration method over existing alternatives include the non-invasive character of the registration, improvement to pre-operative logistics and prevention of additional radiation exposure to the patient. The technique has been implemented in our hospital for dentate facial trauma patients whose maxillary complex is still attached to the cranium, and secondary reconstructive cases. The workflow to obtain a registration splint takes approximately 3–4 h from start of the (bedside) intra-oral scan to completion of the 3D printing process. If an intra-oral scan is not available, impressions can be taken and the cast models can be digitized through (CB)CT imaging. The workflow allows fabrication of a registration splint even in acute trauma patients, if in-house printing facilities are available.

## Conclusion

In this study, a registration method using virtual fiducials on a 3D printed dental splint was presented. The registration accuracy was evaluated in a cadaver study, for optical and EM tracking, and compared to bone-anchored maxillary fiducial registration (optical and EM) and surface-based soft-tissue registration (EM). At the level of the orbital rim, only small differences in the error estimates of bone-anchored fiducial registration and splint-based registration were seen; both outperformed the soft-tissue registration at the infraorbital rim level. The loss of accuracy over distance from the registration area was significantly lower for soft-tissue registration than for any of the point-based registration methods. The virtual fiducial registration method has advantages over bone-anchored fiducials in terms of invasiveness and over existing splint-based methods in terms of fiducial identification and radiation exposure, and offers an excellent alternative for registration in oral and maxillofacial surgery.

## Data Availability

The datasets generated during and/or analysed during the current study are available from the corresponding author on reasonable request.
